# Supplementary material for: Domain prediction with probabilistic directional context
Source: Bioinformatics. 2017 Apr 12;33(16):2471–8. doi: 10.1093/bioinformatics/btx221 (PMC5870623; doi:10.1093/bioinformatics/btx221)
Supplement: Supplementary Data [file supp_btx221.pdf]

Supplement for: Domain prediction with probabilistic directional context

Alejandro Ochoa and Mona Singh

S1 Summary

The results in the main text are based on Pfam 25. Here, we test the context methods using the newer Pfam 30 version with the RevSeq FDR test using UniProt.

S2 Methods

Pfam 30 (16,306 HMMs) provides the Pfam-A.full.uniprot file that corresponds to UniProt 2016_02 (46,974,580 proteins). This file was used to obtain dPUC2's observed family pair counts, CODD's list of certified domain pairs [1], and DAMA's domain information and observed architectures [2]. We used the HMMER 3.1b2 version of `hmmscan` to predict domains (this version is required by Pfam 30). We downloaded the newest UniRef50 version (dated 2017-02-27, 20,905,476 proteins) and randomly selected a subset of 1,000,000 proteins to use in the Pfam 30 version of the RevSeq FDR test, which is otherwise as before [3]. All domain prediction methods were run as described in the main text.

S3 Results

We find that the ranking of all domain prediction methods is consistent between Pfam 30 (Fig. S1) and Pfam 25 (Fig. 3 in the main text), with dPUC2 outperforming its competitors. The FDR ranges of the dPUC2, dPUC1 and CODD predictions is comparable between these Pfam versions, but FDRs are generally higher for DAMA using Pfam 30 than they were when using Pfam 25 (x-axis in Fig. S1 and Fig. 3). All context methods have greater improvements in the number of predicted domains compared to the Standard Pfam 30 than they did for Pfam 25 (y-axis in Fig. S1 and Fig. 3). In particular, dPUC2 with a threshold of $p <$ 1e-4 for candidate domains predicts over 20% more domains than the Standard Pfam 30 (Fig. S1), whereas the improvement was about 15% for Pfam 25 (Fig. 3).

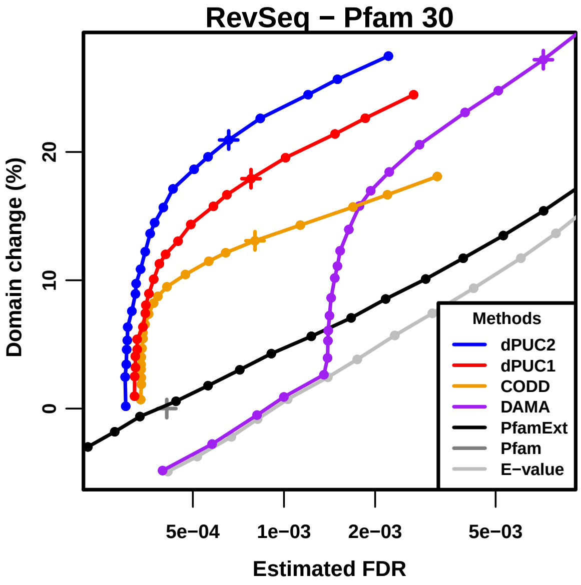

Figure S1: Using the newest Pfam 30 release, dPUC2 predicts more domains than its competitors across a wide range of FDRs, as estimated by the RevSeq test. Details match Fig. 3 in the main text.

References

[1] N. Terrapon et al. "Detection of new protein domains using co-occurrence: application to Plasmodium falciparum". *Bioinformatics* 25(23) (2009), pp. 3077–3083.

[2] J. S. Bernardes et al. "A multi-objective optimisation approach accurately resolves protein domain architectures". *Bioinformatics* (2015), btv582.

[3] A. Ochoa et al. "Beyond the E-Value: Stratified Statistics for Protein Domain Prediction". *PLoS Comput Biol* 11(11) (2015), e1004509.
